# *Chlorella vulgaris* lipid extraction side-stream enhances growth and protein enrichment in novel food *Lemna minor* (duckweed)

**DOI:** 10.3389/fnut.2026.1822150

**Published:** 2026-05-29

**Authors:** Giacomo Fais, Silvia Castelli, Debora Dessì, Giovanni Perra, Nicola Lai, Giacomo Cao, Alessandro Concas

**Affiliations:** 1 Interdepartmental Centre of Environmental Science and Engineering (CINSA), University of Cagliari, Cagliari, Italy; 2 Department of Mechanical, Chemical and Materials Engineering, University of Cagliari, Cagliari, Italy; 3 Department of Life and Environmental Sciences, University of Cagliari, Cagliari, Italy

**Keywords:** bio-based fertilizer, *Chlorella vulgaris*, circular bioeconomy, duckweed, growth stimulation, *Lemna minor*, lipid extraction, microalgal side-stream

## Abstract

**Introduction:**

The transition toward sustainable food systems requires strategies that simultaneously reduce bioprocess residues and enhance nutritional quality. Microalgal biorefineries generate substantial polar side-streams after lipid extraction, which remain largely underutilized despite their high nutrient content.

**Methods:**

Here, we evaluated the polar side-stream generated during lipid extraction of *Chlorella vulgaris*, representing the non-lipid and water-soluble fraction enriched in soluble metabolites and nutrients, as a bio-based fertilizing input for *Lemna minor* (duckweed), a Novel Food. The *Chlorella* Extraction Waste (CEW) was applied in hydroponic cultivation across a defined concentration window, and growth performance was quantified through image-based frond area analysis and kinetic modeling.

**Results:**

Within a non-inhibiting range, the side-stream significantly stimulated duckweed growth, with 0.2 g L^−1^ nearly doubling biomass at day 7 compared to water control. Bench-scale validation against Hoagland solution and a commercial fertilizer demonstrated enhanced protein accumulation (27.1% DW), exceeding the commercial fertilizer treatment and accompanied by reduced carbohydrate allocation, while maintaining balanced pigment profiles, antioxidant activity, and stable lipid levels.

**Discussion:**

The absence of carotenoid overaccumulation and major shifts in antioxidant activity indicates nutrient-enabled protein enrichment without detectable stress-associated metabolic reprogramming. These findings demonstrate that microalgal lipid extraction side-streams can function as nutrient-regime modulators capable of redirecting carbon–nitrogen allocation toward protein-rich biomass, coupling circular bioeconomy valorization with nutritional upgrading of a fast-growing aquatic crop. This work provides a proof-of-concept for integrating biorefinery side-streams into sustainable micro-scale photosynthetic food systems.

## Graphical abstract

**Figure:**
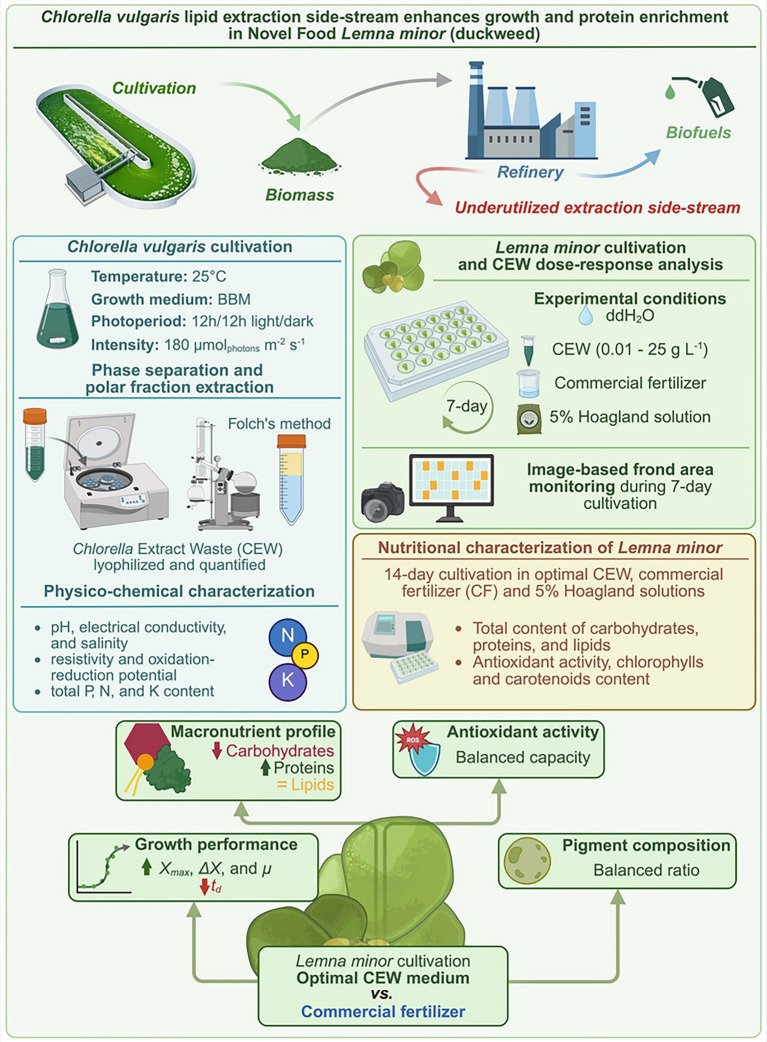

## 1 Introduction

The combined pressures of population growth and climate change are intensifying constraints on food production (1). Sustainable approaches that reduce reliance on synthetic inputs are therefore increasingly needed (2–4). In this context, microalgae and aquatic plants are emerging as promising resources to couple nutritional value with lower environmental impact (5–8). Microalgae have been extensively cultivated for biodiesel production within biorefinery frameworks, where lipid extraction often represents the primary value stream (9). This process generates substantial residual fractions, particularly polar side-streams that retain a significant proportion of the original biomass in the form of soluble proteins, carbohydrates, and micronutrients (10–14).

Despite their biochemical richness, lipid extraction co-products are frequently underutilized or discarded, and current valorization efforts have largely focused on delipidated solid residues. In contrast, for *Chlorella vulgaris*, the polar and water-soluble side-stream generated during solvent-based lipid extraction, representing the non-lipid fraction of the biomass enriched in soluble metabolites and nutrients, remains comparatively underexplored despite its potential for downstream applications. Notably, this fraction does not correspond to a targeted extract, but to a process-derived stream generated through solvent partitioning and phase separation, where compounds partition between phases according to their physicochemical properties.

Recent work has shown that lipid-extracted *Chlorella vulgaris* residues can support downstream biological processes thanks to their retained nitrogenous and carbohydrate fractions, but these studies have largely addressed delipidated solid biomass rather than the aqueous polar side-stream (8, 15–18). This distinction is important, as most previous studies have considered residual biomass as a whole, without resolving or specifically investigating the individual fractions generated during extraction, particularly the aqueous polar phase (19–21). Accordingly, its valorization could reduce bioprocess residues while supporting circular bioeconomy strategies aimed at improving agricultural sustainability and generating added value (9, 22, 23).

Complementary to microalgal biorefinery strategies, fast-growing aquatic crops of the *Lemnaceae* family have attracted increasing scientific and commercial interest (24, 25). Among these, *Lemna minor* is a fast-growing floating freshwater species with biomass doubling times as short as 48 h under optimal conditions (25–29). This growing interest reflects its emerging role as a high-protein crop in controlled cultivation systems. *Lemna minor* exhibits significant physiological plasticity, with variations in growth rate, stress tolerance, and nutrient accumulation reported across clonal lines (26, 30, 31).

Its potential as a sustainable protein source is increasingly recognized. Protein content can reach approximately 45% of dry weight and includes all essential amino acids, comparable to conventional sources such as soy and fish (6, 28). In addition, *L. minor* provides lipids, micronutrients, and bioactive compounds of nutritional relevance for human consumption and food applications, supporting its use in controlled cultivation systems (29, 32). Its phytochemical profile includes antioxidants and anti-inflammatory compounds that may mitigate oxidative stress and support immune function (6, 31, 33). These characteristics are particularly relevant for controlled and closed production systems (34–37).

In recognition of its nutritional value and safety, *Lemna minor* was included in the European Union’s Novel Food Catalogue in 2024 (38), supporting its integration into human diets. Its rapid growth and efficient resource use have also led to its consideration for controlled and closed cultivation systems (39, 40). Despite these promising attributes, optimizing *L. minor* cultivation remains a critical challenge. Chemical fertilizers, although effective in enhancing productivity, may compromise the sustainability of hydroponic systems, pose environmental and health concerns, and significantly increase operational costs, particularly in controlled cultivation settings. Natural alternatives such as biofertilizers are therefore gaining increasing attention (7, 41, 42).

Among these, microalgal biomass-derived products, including extracts and biorefinery side-streams, are promising inputs. They combine readily available nutrients with organic compounds that can support nutrient uptake, stimulate biomass production, and modulate carbon–nitrogen allocation (20, 43, 44). Water-soluble microalgal fractions have been reported to contain compounds of potential agronomic relevance, including readily available nutrients, amino acids, small peptides, organic acids, and other low-molecular-weight metabolites that may contribute to plant growth responses, nutrient uptake, and carbon–nitrogen partitioning (19, 20). However, in most cases these products are obtained through direct extraction of whole biomass (e.g., aqueous or enzymatic extraction), whereas process-derived side-streams generated during lipid extraction have received comparatively limited attention (8, 13, 19–21).

In this context, the polar side-stream obtained after lipid extraction of *Chlorella vulgaris* is expected to retain a substantial proportion of soluble nutrients and metabolites within a process-derived matrix shaped by phase partitioning (12, 13). Accordingly, our working hypothesis is that this polar fraction (CEW) may act as a bio-based input capable of modulating *Lemna minor* growth and biomass composition, primarily through its nutrient content and stoichiometry, while potential contributions of matrix-associated bioactive compounds remain to be clarified (19, 21). In this framework, while stress-based approaches are commonly used to enhance bioactive compounds in photosynthetic foods, nutrient regime modulation may represent a complementary strategy for compositional upgrading, such as protein enrichment, under growth-permissive conditions, without eliciting stress-associated metabolic signatures. This process-derived side-stream is typically treated as a low-value residue, despite containing proteins, carbohydrates, and bioactive compounds that could be repurposed in agriculture (8). Unlike aqueous microalgal extracts obtained by mild maceration, which represent biomass-derived fractions, this material arises from solvent-based extraction workflows and therefore reflects a process-derived composition (44). Reintegrating this side-stream into agricultural systems not only reduces industrial residues but also supports the development of circular bioeconomy strategies in the agri-food sector. To date, no experimental study has tested lipid extraction polar side-streams from microalgae as nutrient amendments in duckweed cultivation, and their effects on growth and biomass composition remain unknown. Therefore, investigating such fractions requires interpreting their composition and functionality as outcomes of biorefinery operations, rather than of targeted extraction strategies. This study evaluates the *Chlorella vulgaris* lipid extraction polar side-stream (CEW) as a biofertilizer for *Lemna minor*, testing the hypothesis that this process-derived polar fraction can modulate growth performance and biomass composition through its nutrient-rich soluble matrix. Overall, this work links microalgal side-stream valorization with the cultivation of a protein-rich aquatic crop, contributing to circular bioeconomy strategies in sustainable agri-food systems.

## 2 Materials and methods

### 2.1 Cultivation of *Chlorella vulgaris*

*Chlorella vulgaris* CCALA 269 (Culture Collection of Autotrophic Organisms, Třeboň, Czech Republic) was cultivated in sterilized 250 mL glass flasks (150 mL of culture) under constant agitation (100 rpm) and a 12 h light/12 h dark photoperiod at 25 °C, with illumination provided by full-spectrum LED grow lights (white 3,000–5,000 K, red 660 nm, far-red 730 nm) at an intensity of 180 μmol photons m^−2^ s^−1^. Cultures were prepared in triplicate, sealed with cotton stoppers to allow gas exchange, and later scaled up to 5 L flasks. Bold’s Basal Medium (BBM) supplemented with bicarbonate was used as growth medium. Growth was monitored daily by optical density (OD) at 650 nm (Genesys 20, Thermo Fisher Scientific, United States) and pH (Basic 20, Crison Instruments, Spain), and a calibration curve was established to correlate OD with dry biomass (Supplementary Figures 1, 2).

### 2.2 Polar fraction extraction (CEW)

During the late exponential growth phase, *Chlorella vulgaris* biomass was harvested by centrifugation (Heraeus Megafuge 1.0R, Thermo Fisher Scientific) at 4000 rpm and 20 °C for 10 min. After centrifugation, the biomass pellet was washed three times with deionized water, resuspended, and centrifuged again under the same conditions to remove residual culture medium components prior to freezing and lyophilization. The pellet was then frozen at −20 °C and lyophilized using a Büchi Lyovapor L-200.

Extraction was performed according to the method of Folch et al. (45) with modifications. A total of 5 g of lyophilized biomass (dry weight, DW) was weighed and transferred into a 500 mL amber glass bottle equipped with a PTFE-lined screw cap, in order to minimize solvent evaporation and light exposure. Extraction was carried out using a chloroform–methanol system (2:1, v/v), preceded by a methanol pre-extraction step (solvent:biomass ratio of 20 mL g^−1^; total methanol volume: 100 mL) to enhance penetration of the polar solvent. Specifically, the biomass was incubated in methanol (100 mL) overnight (approximately 16 h) at 4 °C under gentle agitation (100 rpm) on an orbital shaker to promote solvent diffusion while limiting degradative processes. Cell disruption was subsequently achieved by ultrasonic sonication using a Bandelin Sonoplus HD for 10 min, with samples maintained in an ice bath to prevent overheating and sample degradation. Following sonication, chloroform (200 mL) was added to reach the final chloroform–methanol ratio of 2:1 (v/v). The suspension was then mixed for 1 h using a magnetic stirrer (300 rpm) at room temperature (~22 °C) to ensure thorough solvent–biomass interaction. Separation of the solvent–biomass mixture was achieved by centrifugation at 4000 rpm and 4 °C for 10 min. The supernatant was collected and treated with a 0.88% KCl solution (corresponding to 20% of the total volume, 60 mL), followed by a second centrifugation under the same conditions to induce phase separation. This step yielded a polar (aqueous–methanolic) phase and an apolar (chloroformic) phase. The polar fraction was collected separately, while the apolar phase was discarded as not relevant to the scope of this study.

The residual pellet was subjected to two additional consecutive extraction cycles following the same procedure to maximize recovery. The polar phase obtained from each cycle had an approximate volume of 60 mL. For each cycle, a 1 mL aliquot was dried to constant weight for gravimetric quantification (mg mL^−1^) (Supplementary Figure 3).

The polar fractions obtained from the three extraction cycles were pooled, and organic solvents were removed using a rotary evaporator (Rotavapor R-210, Büchi). The remaining aqueous fraction, hereafter referred to as *Chlorella* extraction waste (CEW), was diluted with deionized water, frozen at −20 °C, and subsequently lyophilized. The lyophilized CEW was then used as a biofertilizer in *L. minor* cultivation experiments.

### 2.3 Physico-chemical characterization of the CEW

The CEW solution was prepared at its optimal concentration for *L. minor* cultivation (0.20 g DW L^−1^), as determined through preliminary dose–response and optimization experiments (see Experimental design section), and analyzed at 20 °C for pH, electrical conductivity (EC), salinity, resistivity, and oxidation–reduction potential (ORP) using a multiparameter probe (HI 98194, Hanna Instruments, United States).

Macronutrients were quantified spectrophotometrically on CEW prepared at the same concentration: total phosphorus (LCK 349, Hach Lange GmbH; digestion 100–120 °C, 15 min; DR6000), total nitrogen (LCK 338, Hach Lange GmbH; 120 °C, 30 min; DR6000), and potassium (H193750, Hanna Instruments; dedicated photometer). In addition, inorganic nitrogen species were determined as nitrate (LCK 340, Hach Lange GmbH) and ammonium (LCK 303, Hach Lange GmbH), while nitrite was analyzed and found to be below the limit of quantification. Organic acids were quantified using cuvette tests (LCK 365, Hach Lange GmbH) and expressed as acetic acid equivalents.

### 2.4 *Lemna minor* cultivation

*Lemna minor* was obtained from a local aquatic plant supplier and subsequently established as a vegetatively propagated indoor laboratory culture at the Interdepartmental Centre of Environmental Science and Engineering (CINSA), University of Cagliari, Italy, prior to experimental use. Plants were maintained in a controlled-environment incubator (FOC 120I, VELP Scientifica, Italy) at 24 °C under 110 μmol photons m^−2^ s^−1^ with a 12 h light/12 h dark photoperiod. Mother cultures were kept in plastic containers with 5% Hoagland’s nutrient solution prepared following Hothem et al. (46). Medium was renewed as needed to keep plants in a vegetative, exponentially growing state.

### 2.5 Image-based growth analysis and kinetic parameters of *Lemna minor*

The growth of *Lemna minor* was monitored using an image-based approach, with frond area employed as the primary growth descriptor. This method was selected due to the macroscopic and floating nature of *L. minor* and provides a robust and reproducible proxy for biomass estimation in duckweed-based systems.

Cultures were photographed at defined time intervals using a fixed imaging setup to ensure consistent distance, illumination, and background conditions. Images were analyzed using ImageJ software (NIH, Bethesda, MD, United States). Prior to analysis, spatial calibration was performed using the known diameter of the cultivation wells (1.6 cm). Fronds were segmented from the background by applying color thresholding and individual regions of interest (ROIs) were automatically or manually selected as needed. The total projected frond area was calculated as the sum of the areas of all ROIs and expressed in cm^2^. Growth was reported as absolute frond area and as percentage increase relative to the initial area on day 0.

Due to the small size of *Lemna minor* fronds, repeated dry weight measurements were not feasible without destructive sampling. Growth was therefore assessed non-destructively using image-based frond area (cm^2^) calibrated against fresh weight (FW, mg), with dry weight determined only at the experimental endpoint. Independent *L. minor* samples covering the full range of observed frond areas were gently blotted to remove excess surface water and weighed immediately to determine FW. The corresponding frond areas were quantified by image analysis as described above, and linear regression was used to derive the calibration curve (Supplementary Figure 5). This calibration curve was subsequently applied to convert frond area values into estimated fresh biomass for all growth experiments.

### 2.6 Growth parameters

Fresh biomass values were estimated from the frond area–fresh weight (FW) calibration curve (Supplementary Figure 5) were used to calculate growth parameters. The maximum biomass concentration (*X_max_*, mg FW L^−1^) was defined as the highest fresh biomass reached during the cultivation period. Average biomass productivity (*ΔX*, mg FW L^−1^ d^−1^) was calculated as:

$$\mathrm{\Delta X}=\frac{X_{\max}}{t_{\max}}$$ (1)

Where, *t_max_* (d) is the time required to reach *X_max_*. The specific growth rate (*μ*, d^−1^) was calculated during the exponential growth phase according to:

$$\mu =\frac{\ln(X_{2})-\ln(X_{1})}{t_{2}-t_{1}}$$ (2)

Where, *X_1_* and *X_2_* represent fresh biomass (mg FW L^−1^) at times *t_1_* and *t_2_*, respectively. The duplication time (*t_d_*, h) was calculated as:

$$t_{d}=\frac{\ln(2)}{\mu }$$ (3)

Dry weight (DW) measurements were performed exclusively on endpoint biomass and were used solely for nutritional and compositional analyses, not for growth kinetics.

### 2.7 Experimental design

**Figure 1:**
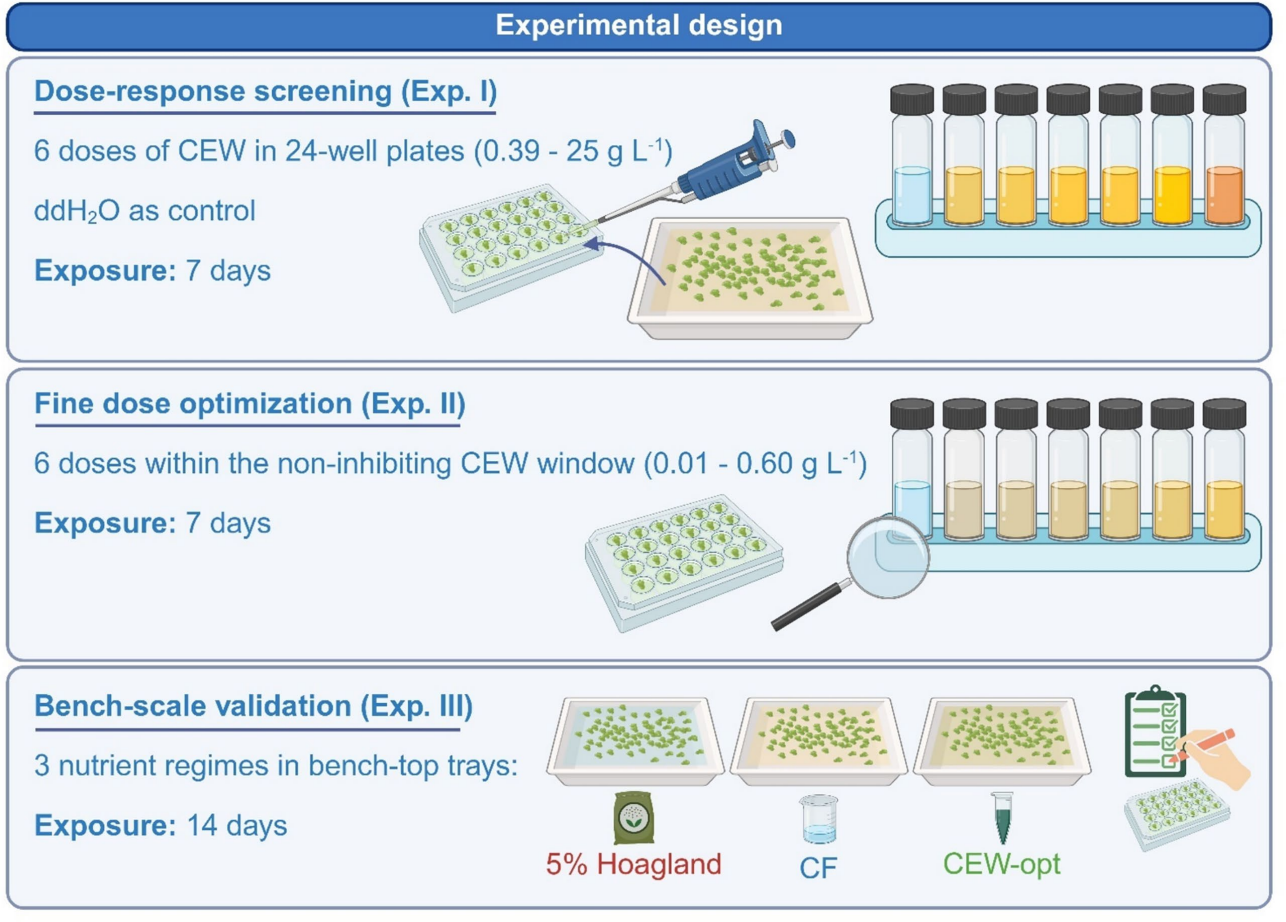
Schematic representation of the experimental workflow. The study was structured into three consecutive phases: dose–response screening (Exp. I), fine dose optimization (Exp. II), and bench-scale validation (Exp. III). Graphical figure was created with BioRender.com.

The experimental workflow consisted of three consecutive phases (Figure 1). A preliminary range-finding assay was conducted in 24-well plates to identify a concentration window not associated with growth inhibition and potentially promoting *Lemna minor* growth when exposed to CEW (0.39–25 g L^−1^) in ddH_2_O; ddH_2_O water as negative control; data not shown, as this preliminary step was used exclusively to define the concentration range for subsequent experiments.

Based on these results, a fine dose-optimization experiment was performed by testing CEW at 0.60, 0.50, 0.40, 0.30, 0.20, and 0.01 g L^−1^ over a 7-day cultivation period. The optimal concentration was selected based on quantitative growth descriptors derived from image-based analysis. Owing to the limited cultivation area of the multi-well format, biomass expansion was constrained and cultivation could not be extended beyond 7 days; nevertheless, this timeframe was sufficient to reliably capture growth kinetics and to discriminate among treatments based on quantitative image-derived growth parameters.

Finally, bench-scale validation was carried out in tray-based systems using 5% Hoagland solution as the base medium, comparing three nutrient regimes: 5% Hoagland alone (control), a commercial fertilizer (ABA Aquarium, applied according to the manufacturer’s instructions), and CEW at the optimal concentration identified previously. This configuration enabled extended cultivation and sufficient biomass accumulation for downstream compositional analyses. Cultures were monitored every 48 h for 14 days, after which endpoint biomass was harvested for nutritional characterization.

The primary endpoint was the percentage increase in total frond area at day 7 relative to day 0. Secondary endpoints included fresh biomass, dry biomass, maximum area, average biomass productivity (*ΔX*), specific growth rate (*μ*), duplication time (*t_d_*), and nutritional parameters (proximate composition, pigments, and antioxidant activity).

The experimental units were individual wells (two fronds in 3 mL) and fixed area tray compartments seeded with a standardized inoculum. Unless otherwise stated, experiments were conducted with n = 4 biological replicates. Treatments were randomized, edge wells were filled with sterile water, and plates and trays were treated as blocks under identical environmental conditions.

### 2.8 Nutritional analysis of *Lemna minor*

*Lemna minor* was grown under three nutrient regimes during the bench-scale tray experiment: (i) 5% Hoagland solution (control), (ii) a commercial fertilizer (CF) (ABA Aquarium, Aquaristica srl; applied according to the manufacturer’s instructions), and (iii) CEW at the optimal concentration. At the end of the 14-day cultivation, plant material from each tray compartment was collected, gently blotted on absorbent paper to remove excess surface water, frozen at −20 °C for 24 h, and subsequently freeze-dried to obtain dry biomass for analysis. All assays were performed in triplicate on aliquots from the same lyophilized batch per treatment, corresponding to three independent biological replicates.

Total carbohydrate content was determined using the colorimetric method of Dubois et al. (47). Two milligrams of freeze-dried sample were extracted in phosphate buffer saline (PBS, 20 mM, pH 7.4) by vortexing and sonication. The extracts were reacted with phenol (5% w/v) and concentrated sulfuric acid, and absorbance was measured at 490 nm using glucose as an external standard. Results were expressed as g per 100 g dry weight (DW) ± standard deviation.

Protein content was determined using a modified Lowry et al. (48) method. Two milligrams of freeze-dried sample were suspended in PBS, mechanically disrupted, and sonicated. Extracts were treated with NaOH (1 N) and incubated at 100 °C, followed by the addition of alkaline copper solution and Folin–Ciocalteu reagent. Absorbance was measured at 750 nm, and protein concentration was calculated using bovine serum albumin (BSA) as a standard. Results were expressed as g per 100 g DW ± standard deviation.

Total lipid content was quantified following a modified Bligh and Dyer (49) extraction protocol. Five milligrams of lyophilized biomass were extracted using PBS and methanolic NaOH, followed by sonication, heating, and centrifugation. Lipids were extracted with a chloroform–methanol mixture and potassium chloride solution. After phase separation, the chloroform phase was dried and subjected to a vanillin–phosphoric acid colorimetric reaction (50). Absorbance was measured at 530 nm, and lipid content was expressed as g per 100 g DW (canola oil equivalents) ± standard deviation.

### 2.9 Pigments and antioxidant activity

Total chlorophylls and carotenoids were determined according to Zavrel et al. (51). Pellets obtained after centrifugation (10,000 rpm, 4 °C) of methanolic extracts were resuspended in neutralized methanol and incubated at 4 °C for 24 h. Samples were subsequently homogenized by vortexing and sonication, centrifuged again, and analyzed spectrophotometrically. Absorbance was measured at 665 nm (chlorophylls), 470 nm (carotenoids), and 720 nm for baseline correction. Pigment concentrations were calculated using the equations proposed by Ritchie (52) and Wellburn (53) and expressed as mg g^−1^ dry weight (DW) ± standard deviation.

Antioxidant activity was assessed using the DPPH radical scavenging assay (54). Five milligrams of lyophilized biomass were extracted in methanol and centrifuged, and 50 μL of the resulting extract was mixed with 2 mL of a DPPH methanolic solution (50 μmol). After incubation for 60 min in the dark, absorbance was measured at 517 nm. Trolox was used as an external standard, and results were expressed as mmol Trolox equivalents (TEAC) g^−1^ DW ± standard deviation.

### 2.10 Statistical analysis

Data were expressed as mean ± standard deviation (SD). Statistical analyses were performed using one-way analysis of variance (ANOVA) followed by Tukey’s *post hoc* test for multiple comparisons, with *p* < 0.05 considered statistically significant. The experimental unit was the individual well or tray compartment, and analyses were conducted on biological replicates. Statistical analyses and data visualization were performed using GraphPad Prism 9.0 (GraphPad Software, San Diego, CA, United States).

## 3 Results and discussion

### 3.1 Production of *Chlorella vulgaris* biomass for the recovery of the polar extract (CEW)

The growth of *C. vulgaris* cultures was monitored over the experimental period by measuring optical density (OD) at 650 nm and dry biomass concentration. Cultures exhibited a progressive increase in biomass, reaching the stationary phase after 11 days, with a maximum dry biomass concentration of 1.16 ± 0.05 g L^−1^ (Supplementary Figures 1, 2). This cultivation timeframe ensured the production of sufficient biomass for downstream extraction of the polar fraction (CEW).

The CEW was obtained from 5 g dry weight (DW) of freeze-dried *C. vulgaris* biomass using a modified Folch extraction protocol (Section 2.2), selected to reproduce at laboratory scale a conventional solvent-based process typically employed for microalgal oil and lipid extraction, thereby generating a polar side-stream representative of the aqueous fraction arising from solvent-based lipid extraction and phase partitioning (12, 45, 55).

To maximize recovery, three consecutive extraction cycles were performed on the same biomass, each yielding approximately 60 mL of polar phase. Gravimetric analysis of 1 mL aliquots dried to constant weight revealed residue concentrations of 39.97 ± 1.35, 20.80 ± 1.24, and 5.01 ± 0.71 mg mL^−1^ for the first, second, and third extraction cycles, respectively. Based on the recovered volume per cycle, the corresponding dry masses were 2,398 ± 81 mg (cycle 1), 1,248 ± 74 mg (cycle 2), and 301 ± 43 mg (cycle 3). When normalized to the initial biomass, these values corresponded to yields of 479.6 ± 16.2 mg g^−1^ DW (47.96 ± 1.62% DW), 249.6 ± 14.9 mg g^−1^ DW (24.96 ± 1.49% DW), and 60.2 ± 8.6 mg g^−1^ DW (6.02 ± 0.86% DW), respectively. The cumulative recovery of polar material across the three extraction cycles was 3.95 ± 0.19 g, corresponding to 789.3 ± 38 mg g^−1^ DW or 78.93 ± 3.8% of the initial biomass. The majority of the polar fraction (≈ 48%) was recovered during the first extraction cycle, while the first two cycles together accounted for approximately ≈ 73% of the total recovered material (Supplementary Figure 3 and Table 1).

**Table 1:** Yield of the polar fraction (CEW) from *Chlorella vulgaris* biomass obtained in three sequential Folch extraction cycles.

Cycle	Residue concentration [mg mL^−1^]	Polar phase volume [mL]	Mass recovered [mg]	Yield of polar fraction [% DW]
1	39.97 ± 1.35	60	2,398 ± 81	47.96 ± 1.62
2	20.80 ± 1.24	60	1,248 ± 74	24.96 ± 1.49
3	5.01 ± 0.71	60	301 ± 43	6.02 ± 0.86
Total	–	–	3,947 ± 194	78.93 ± 3.8

Residue concentration refers to the dry weight content in 1 mL of polar phase (mean ± SD, *n* = 4). Yield values are expressed per gram of initial dry biomass (DW) and as a percentage of the initial DW.

The cumulative recovery reported here refers to the gravimetric recovery of the dry residue in the polar stream after solvent removal, reflecting the transfer of the non-lipid, water-soluble biomass fraction rather than a chemically defined extract. Accordingly, CEW should be interpreted as a process-derived fraction, consistent with the Folch extraction framework, in which the polar phase contains the non-lipid fraction of the biomass following phase partitioning (45). Considering the biochemical composition of *C. vulgaris*, typically contain approximately 10–25% lipids on a dry weight basis, this level of recovery is consistent with mass balance expectations, as proteins, carbohydrates, and soluble constituents account for the majority of the biomass once lipids are removed. The observed recovery (~79%) therefore aligns with values commonly reported for *Chlorella*-based systems following lipid extraction (12, 13).

Comparable recoveries of water-soluble material have been reported for *Chlorella* and other green microalgae under solvent-based extraction conditions, supporting the transfer of a substantial share of non-lipid biomass into the polar phase (12, 13). Similar trends have also been observed in microalgal biomass solubilization studies, where disruption processes release intracellular material into the aqueous fraction (56).

From an industrial perspective, solvent-based lipid extraction generates a substantial polar residue as a major by-product stream in microalgal biorefineries. This fraction is increasingly regarded as a valuable secondary resource rather than waste, due to its content of nitrogenous compounds, carbohydrates, and mineral nutrients (57). Consistently, the non-lipid fraction of *C. vulgaris* is enriched in nitrogen-rich biomolecules, soluble carbohydrates, phosphorylated metabolites, and minerals, resulting in a nutrient-dense aqueous phase. From a circular bioeconomy perspective, this side-stream therefore represents a potential nutrient reservoir that may be repurposed in downstream photosynthetic systems.

### 3.2 Evaluation of fertilizing properties of CEW

#### 3.2.1 Physico-chemical parameters

To enable a like-for-like comparison across treatments, pH, electrical conductivity (EC), salinity, resistivity, and oxidation–reduction potential (ORP) were determined at 20 °C for CEW (0.2 g DW L^−1^), Hoagland’s solution at 5% strength, and the commercial fertilizer (CF). Results are reported as mean ± SD (*n* = 3) in Table 2.

**Table 2:** Physicochemical parameters of CEW, Hoagland 5%, and commercial fertilizer (CF) measured at 20 °C.

Parameter	Hoagland 5%	Commercial fertilizer	CEW (0.2 g DW L^−1^)
pH	5.41 ± 0.01	5.47 ± 0.01	6.83 ± 0.02
Electrical conductivity [μS cm^−1^]	198.7 ± 1.5	25.2 ± 0.2	372.0 ± 5.0
Salinity [ppm]	90.8 ± 1.2	12.2 ± 0.3	177.7 ± 2.1
Resistivity [kΩ·cm]	5.10 ± 0.05	37.40 ± 0.13	2.69 ± 0.03

Data values are mean ± SD (*n* = 3).

The CEW solution exhibited a near-neutral pH (6.83 ± 0.02), higher than that of both Hoagland 5% and the commercial fertilizer, which showed mildly acidic values (pH ≈ 5.4–5.5). This pH range falls within the tolerance interval commonly reported for *L. minor* cultivation under hydroponic conditions and does not require external buffering (58). Electrical conductivity and salinity were higher in CEW than in the commercial fertilizer and comparable to or higher than those of Hoagland 5%, reflecting the presence of a greater pool of soluble ionic species transferred into the polar phase during extraction and phase partitioning. Consistently, CEW displayed lower resistivity, indicative of higher ionic strength.

#### 3.2.2 Nutrient availability

At the tested concentration (0.2 g DW L^−1^), CEW provided higher concentrations of total nitrogen, phosphorus, and potassium compared to both the commercial fertilizer (CF) and the 5% Hoagland solution (Table 3).

**Table 3:** Macronutrient content of CEW, Hoagland 5% solution, and commercial fertilizer (CF).

Treatment	Total N [mg L^−1^]	Total P [mg L^−1^]	Total K [mg L^−1^]	N: P: K Ratio^*^
CEW (0.2 g DW L^−1^)	38.42 ± 0.51	15.26 ± 0.22	41.17 ± 0.39	1: 0.40: 1.07
Hoagland 5%^1^	10.50^calc.^	1.55 ^calc.^	11.72^calc.^	1: 0.15: 1.12
Commercial fertilizer^2^	4.12 ± 0.05	<0.1 (LOQ)	8.02 ± 0.12	1: <0.03: 1.95

CEW and CF values are mean ± SD (*n* = 3). ^1^Hoagland 5% values were calculated (^calc.^) from the full-strength formulation and adjusted to 5% dilution (not measured). ^2^ABA Aquarium was prepared at the recommended dose (10 mL per 100 L water) and analyzed using spectrophotometric/photometric kits. For CF, phosphorus, <LOQ indicates concentration below the limit of quantification. ^*^Normalized to *N* = 1.

In particular, total nitrogen and potassium concentrations in CEW were approximately three- to four-fold higher than those supplied by Hoagland 5%, while phosphorus availability was markedly higher than in both reference treatments, remaining below the limit of quantification in the commercial fertilizer. When normalized to nitrogen, CEW exhibited an N: P: K ratio of 1:0.40:1.07, clearly distinct from those of Hoagland 5% and commercial fertilizer (CF). This profile reflects the complex nutrient matrix derived from microalgal biomass, where nitrogen and phosphorus are associated with soluble organic constituents, while potassium is mainly present as a freely soluble intracellular cation. In addition, the extraction workflow may contribute residual potassium salts to the polar fraction.

Organic acids were also detected (58 mg L^−1^), indicating the presence of low molecular weight soluble compounds. Consistent with previous studies, such fractions may include bioavailable organic molecules relevant for plant nutrition (e.g., hormone-like substances such as auxin- and cytokinin-like compounds) (59–62). Consistently with this interpretation, analysis of nitrogen forms indicated that inorganic nitrogen (NO_3_^−^-N = 1.98 mg L^−1^; NH_4_^+^-N < 2 mg L^−1^; NO_2_^−^ below the limit of quantification) represented only a minor fraction relative to total nitrogen (38.42 ± 0.51 mg L^−1^), indicating that nitrogen in CEW is predominantly present in organic forms.

### 3.3 Growth performance of *Lemna minor* under CEW treatment

Growth performance of *L. minor* was evaluated through three sequential experiments (Figure 1): a preliminary range-finding dose–response assay (Exp. I), a fine dose-optimization experiment (Exp. II), and a bench-scale validation under comparative nutrient regimes (Exp. III). Growth was monitored by image-based frond area analysis and expressed as estimated fresh biomass, complemented by kinetic descriptors (Equations 1–3), including maximum biomass (*X_max_*), average biomass productivity (*ΔX*), specific growth rate (*μ*), and duplication time (*t_d_*).

#### 3.3.1 Dose–response screening (Exp. I)

A preliminary range-finding assay was conducted by exposing *L. minor* to a wide range of CEW concentrations (25 → 0.39 g L^−1^) in ddH_2_O, with ddH_2_O water as control. This screening identified a non-inhibiting concentration window in which CEW exerted a growth-promoting effect, while higher concentrations induced growth inhibition. Based on these observations, six concentrations within the non-inhibiting window were selected for subsequent fine optimization.

#### 3.3.2 Fine dose optimization (Exp. II)

**Figure 2:**
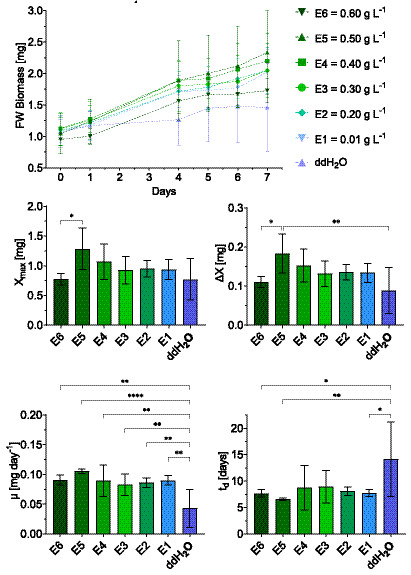
Growth performance of *Lemna minor* under ddH_2_O or different concentrations of CEW over 7 days. Kinetic descriptors: *X_max_*, *ΔX*, *μ*, and *t_d_*. Marks (*) refer to statistical difference between groups: *p < 0.05, **p < 0.01, ***p < 0.001, ****p < 0.0001. Data values are mean ± SD (n = 6) and are based on the calibration curve reported in Supplementary Figure 5.

Six CEW concentrations (0.60, 0.50, 0.40, 0.30, 0.20, and 0.01 g L^−1^) were evaluated over a 7-day cultivation period (Figure 2 and Supplementary Figure 4). The 7-day multiwell assay was designed as a screening and dose-optimization step. Given the rapid growth rate of *L. minor*, this timeframe was sufficient to capture exponential growth; however, biomass expansion in the multiwell format is primarily constrained by the available surface area, leading to density-dependent limitations as fronds progressively occupy the culture space (63, 64). All treatments within this range supported *L. minor* growth relative to water control, but clear dose-dependent differences were observed in both biomass accumulation and kinetic parameters.

Among the tested concentrations, CEW at 0.20 g L^−1^ (CEW-opt) resulted in the highest growth performance, yielding a near-doubling of fresh biomass at day 7 compared to the water control (*p* < 0.05). This treatment also exhibited the highest values of *X*_max_ and ΔX, along with a significantly increased specific growth rate (μ) and a corresponding reduction in duplication time (*t*_d_) relative to the control. At concentrations above CEW-opt (≥0.30 g L^−1^), growth performance progressively declined, as evidenced by lower μ values and longer duplication times, despite the absence of visible phytotoxic symptoms.

This pattern suggests that supra-optimal CEW concentrations may impose physicochemical and metabolic constraints that limit growth efficiency rather than causing acute toxicity. This interpretation is consistent with the bell-shaped dose–response observed, which is characteristic of nutrient-driven systems operating within a narrow optimal window. At supra-optimal concentrations, growth limitation may arise from combined effects, including increased ionic strength and osmotic imbalance affecting water uptake and cellular homeostasis, excess nutrient availability leading to reduced nutrient use efficiency and metabolic imbalance, and alterations in carbon–nitrogen partitioning due to saturation of nitrogen assimilation pathways (65–67). In addition, the accumulation of soluble organic compounds and salts in the medium may contribute to suboptimal physiological conditions. Such responses align with the broader duckweed literature, which highlights the sensitivity of *Lemna* species to medium composition and indicates that phytotoxic effects may occur prior to the onset of visible damage (68–71).

The increased ionic strength associated with CEW suggests that its application represents a mild physicochemical perturbation relative to diluted mineral formulations. However, at 0.2 g DW L^−1^, this perturbation remained within the physiological tolerance range of *L. minor*, as no visible growth impairment, chlorosis, or morphological alteration was observed during cultivation. This indicates that CEW operates within an ionic range compatible with normal growth performance, without triggering overt osmotic stress under the tested conditions. Consistently, *L. minor* has been reported to tolerate relatively wide ranges of water chemistry, including moderate increases in electrical conductivity and salinity, provided that extreme osmotic thresholds are not exceeded (58, 63, 64). Such an increase in ionic strength likely reflects the transfer of intracellular soluble salts and charged metabolites into the polar phase during extraction and phase partitioning, contributing to a more complex aqueous chemical environment compared to purely mineral formulations.

In addition to increased ionic strength, optical effects associated with CEW coloration could potentially contribute to growth limitation at supra-optimal concentrations (72). However, given that *L. minor* is a floating species with photosynthetically active fronds exposed directly at the air–water interface, light interception primarily occurs at the frond surface, where photosynthetically active tissues are directly exposed to the incident light, thereby limiting the influence of bulk medium optical properties on light availability (63, 73). Accordingly, the observed bell-shaped dose–response, together with the absence of visible photoinhibition or pigment imbalance at CEW-opt, indicates that concentration-dependent physiological effects represent the primary driver of growth dynamics under CEW treatment, while light attenuation is unlikely to be a dominant factor within the effective concentration range. These findings confirm that CEW acts as a growth-promoting input only within a defined concentration range, supporting the importance of dosage optimization when repurposing nutrient-rich, process-derived side-streams for plant cultivation.

#### 3.3.3 Bench-scale validation (Exp. III)

The growth-promoting effect of CEW-opt was further validated in a bench-scale tray experiment using 5% Hoagland solution as the base medium. Three nutrient regimes were compared: Hoagland 5% alone (control), a commercial fertilizer (CF), and CEW applied at CEW-opt (0.20 g L^−1^).

**Figure 3:**
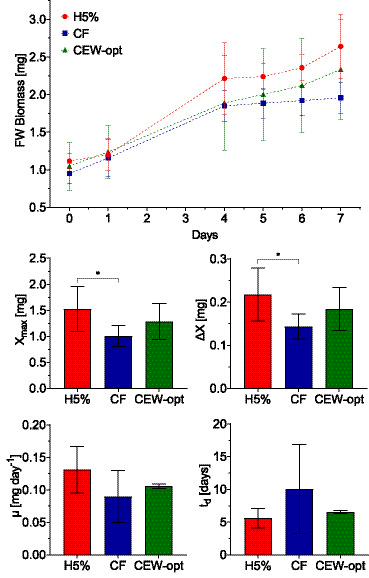
Growth performance of *L. minor* under Hoagland 5% solution (H5%), commercial fertilizer (CF) and CEW at the optimal dose (CEW-opt, 0.20 g L^−1^) over 7 days. Kinetic descriptors: *X_max_*, *ΔX*, *μ*, and *t_d_*. Marks (*) refer to statistical difference between groups: *p < 0.05. Data values are mean ± SD (*n* = 6) and are based on the calibration curve reported in Supplementary Figure 5.

Across the 7-day monitoring period, *L. minor* cultivated under CEW-opt displayed consistently higher biomass accumulation compared to both reference treatments (Figure 3). Growth trajectories diverged from day 4–5 onward, with CEW-opt showing significantly higher *X_max_* and *ΔX* values relative to the commercial fertilizer (*p* < 0.05), while remaining comparable to or exceeding those observed under Hoagland 5%.

Kinetic analysis further indicated that CEW-opt supported efficient growth dynamics, with *μ* values comparable to Hoagland 5% and higher than those obtained with the commercial fertilizer, and duplication times remaining within the optimal range for *L. minor* cultivation. No visible signs of phytotoxicity or morphological alterations were observed under CEW-opt throughout the experimental period. Minor differences in kinetic parameters observed between the multi-well (Exp. II) and tray (Exp. III) setups likely reflect scale-dependent microenvironmental factors, including differences in surface-to-volume ratio, light distribution, and gas exchange dynamics, rather than intrinsic changes in the effect of the CEW treatment. Additionally, the absence of serial dry weight measurements represents a limitation of the study and should be considered when interpreting biomass estimates.

### 3.4 Nutritional composition of *Lemna minor* biomass

**Figure 4:**
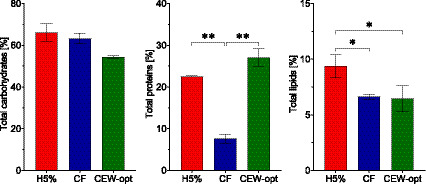
Nutritional composition of *Lemna minor* biomass at day 14 under Hoagland 5% solution (H5%), commercial fertilizer (CF) and CEW at the optimal dose (CEW-opt, 0.20 g L^−1^). Marks (*) refer to statistical difference between groups: *p < 0.05, **p < 0.01. Data values are mean ± SD (*n* = 3).

At day 14, protein, carbohydrate, and lipid contents fell within the ranges commonly reported for actively growing and nutrient-sufficient *L. minor* cultures (29, 74). However, the distinct fertilization regimes applied in the present study resulted in clear differences in biomass composition, indicating that nutrient source and availability modulated macronutrient allocation patterns (Figure 4).

Similar nutrient-driven shifts in proximate composition have been documented under controlled nitrogen and phosphorus regimes, highlighting the compositional plasticity of *L. minor* in response to nutrient supply (75). Specifically, CEW-opt treatment resulted in a marked increase in protein content compared to the commercial fertilizer, with protein levels reaching 27.06 ± 2.08% DW versus 7.62 ± 1.05% DW under CF (*p* < 0.05). Protein content under CEW-opt was also higher than that measured in plants cultivated with Hoagland 5% (22.54 ± 0.13% DW), although the magnitude of this difference was smaller. This protein enrichment under CEW-opt is consistent with the greater nitrogen availability supplied by the CEW solution (Table 3), which likely enhanced nitrogen uptake and assimilation into amino acids and structural proteins. In duckweeds, a substantial fraction of leaf nitrogen is allocated to photosynthetic proteins, including Rubisco and associated enzymatic machinery; therefore, increased nitrogen supply typically translates into higher protein accumulation under non-limiting growth conditions. Conversely, CEW-opt biomass displayed lower total carbohydrate content relative to both reference treatments, indicating a redistribution of carbon toward nitrogen-rich compounds (Figure 4). Such shifts in macronutrient allocation are well documented in duckweeds, where nitrogen availability strongly regulates carbon–nitrogen balance, promoting protein synthesis at the expense of carbohydrate accumulation (58, 63). Taken together, these compositional changes are consistent with nutrient-driven responses associated with higher nitrogen availability under CEW treatment.

Total lipid content in CEW-opt plants was comparable to that observed under the commercial fertilizer and lower than that measured under Hoagland 5%, indicating that protein enrichment was not accompanied by lipid accumulation. This response agrees with previous findings showing that lipid accumulation in *L. minor* is typically associated with nitrogen limitation or stress conditions rather than enhanced nitrogen availability (58, 64). The sum of proteins, carbohydrates, and lipids did not account for the total dry weight, reflecting the inherent compositional complexity of duckweed biomass. In *Lemna* species, a significant fraction of dry matter is commonly represented by ash (often 10–20% DW under nutrient-replete conditions), together with structural polysaccharides and minor metabolites not quantified in the present assays (58, 64).

In this respect, CEW should be interpreted primarily as a process-derived nutrient regime rather than a targeted biostimulant extract. Unlike conventional mineral fertilizers, CEW provides nutrients within a complex matrix in which a substantial fraction of nitrogen is present in organic forms and is accompanied by soluble metabolites such as amino acids and organic acids. Although these components may influence nutrient uptake dynamics, their specific contribution cannot be resolved within the present experimental design. Overall, the observed compositional changes are consistent with a nutrient-driven response, likely associated with the higher nitrogen availability and distinct nutrient stoichiometry provided by CEW, which are known to regulate growth rates and carbon–nitrogen partitioning in duckweeds.

**Figure 5:**
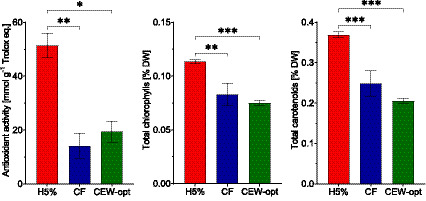
Antioxidant activity and pigment composition of *Lemna minor* biomass at day 14 under Hoagland 5% solution, commercial fertilizer (CF) and CEW at the optimal dose (CEW-opt, 0.20 g L^−1^). Marks (*) refer to statistical difference between groups: *p < 0.05, **p < 0.01, ***p < 0.001. Data values are mean ± SD (*n* = 3).

In order to contextualize these compositional changes within the broader physiological response of the plant, antioxidant activity and photosynthetic pigment content were assessed. CEW-opt treatment resulted in intermediate antioxidant activity compared to the reference treatments, with DPPH radical scavenging activity significantly higher than that measured in CF-grown plants, while remaining lower than that observed under Hoagland 5% conditions. This pattern aligns with previous studies indicating that antioxidant capacity in duckweeds is modulated by nutrient availability and photosynthetic performance, rather than being exclusively triggered by stress responses (63, 64). Similarly, photosynthetic pigment content under CEW-opt was maintained at levels comparable to or slightly lower than those observed in Hoagland-grown biomass and higher than or similar to those obtained with the commercial fertilizer. Chlorophyll content under CEW-opt was lower than that measured under Hoagland 5% but comparable to CF, whereas total carotenoid content showed intermediate values between the two reference treatments (Figure 5). Comparable pigment profiles have been reported in *Lemna* species cultivated under nutrient conditions that sustain active growth without inducing nutrient limitation or stress-associated pigment accumulation (64, 76).

Consistently, the absence of pronounced carotenoid overaccumulation or strongly elevated antioxidant activity under CEW-opt, together with the lack of lipid enrichment observed in proximate composition, suggests that the treatment did not induce detectable stress-associated metabolic reprogramming at the cultivation endpoint, based on the indicators assessed. In stress-driven nutritional enhancement strategies, increased carotenoid pools and antioxidant capacity often reflect reactive oxygen species (ROS) mitigation. In contrast, the intermediate physiological profiles observed here are consistent with a metabolically balanced and growth-oriented state maintained under adequate nutrient supply. However, these indicators provide only indirect evidence, and additional physiological markers (e.g., chlorophyll fluorescence parameters, ROS levels, or membrane integrity indicators) would be required to conclusively assess potential stress responses.

### 3.5 Future perspectives for circular bioeconomy

From a circular bioeconomy perspective, the valorization of microalgal biorefinery side-streams represents a promising yet still emerging strategy that requires careful contextualization. Microalgal biomass derived from species such as *Chlorella* spp. typically contains moderate lipid fractions under nutrient-replete conditions, leaving a substantial proportion of non-lipid material following extraction processes (12, 13). This fraction, including both solid and soluble components depending on the extraction pathway, constitutes a potentially valuable yet underutilized resource. Microalgal biomass production remains cost-intensive, with reported costs typically around 2.0 $ kg^−1^ dry biomass under current technologies (77). In this context, unvalorized residual streams contribute to downstream processing and disposal costs, whereas their integration into secondary value chains has been proposed as a strategy to improve process economics and reduce environmental impact (78, 79).

Accordingly, the reuse of such side-streams in agri-food systems should be regarded as a proof-of-concept rather than an established or scalable solution, as current implementations remain largely confined to laboratory or pilot-scale systems. Residual biomass after lipid extraction is widely recognized as a key co-product in microalgal biorefineries; however, the present study specifically addresses a process-derived polar fraction rather than the residual biomass as a whole (10, 12, 13, 80, 81). Therefore, its potential contribution to waste reduction, nutrient recycling, and sustainable food production remains context-dependent and not yet generalizable across different production systems (9, 23). From a scalability standpoint, key challenges include process optimization, compositional standardization, and validation under realistic cultivation conditions. Variability in biomass composition across strains, growth conditions, and extraction processes may significantly affect reproducibility and limit direct translation into operational systems (13).

While the present study demonstrates the feasibility of using a lipid extraction polar fraction as a nutrient regime modulator to enhance growth and protein accumulation in *L. minor*, its applicability beyond controlled experimental conditions remains to be established. Future efforts should therefore focus on ensuring compositional consistency, defining robust operating conditions, and disentangling the relative contribution of nutrient availability and matrix-associated bioactive components, for example through the use of nutrient-matched controls and fractionation approaches.

## 4 Conclusion

This study demonstrates that the polar side-stream generated during lipid extraction of *Chlorella vulgaris* can be repurposed as a biofertilizing input for *L. minor*. Within a defined non-inhibiting concentration range, CEW supported biomass accumulation and significantly increased protein content. The observed response was consistent with nutrient-driven modulation, promoting carbon–nitrogen reallocation toward protein-rich biomass without evidence of stress-associated metabolic responses or growth penalties. These findings highlight the potential of microalgal side-streams as nutrient sources for controlled cultivation systems.

## Data Availability

The original contributions presented in the study are included in the article/Supplementary material, further inquiries can be directed to the corresponding authors.
