# Supplementary material for: Chlorella vulgaris lipid extraction side-stream enhances growth and protein enrichment in novel food Lemna minor (duckweed)
Source: Front Nutr. 2026 May 29;13:1822150. doi: 10.3389/fnut.2026.1822150 (PMC13259828; doi:10.3389/fnut.2026.1822150)
Supplement: Supplementary file 1 [file Data_Sheet_1.PDF]

Supplementary Material

Chlorella vulgaris lipid extraction side-stream enhances growth and protein enrichment in Novel Food *Lemna minor* (duckweed)

Giacomo Fais[1,2,*], Silvia Castelli[2], Debora Dessì[3], Giovanni Perra[2], Nicola Lai[1,2], Giacomo Cao[1,2], and Alessandro Concas[1,2,*]

[1] Interdepartmental Centre of Environmental Science and Engineering (CINSA), University of Cagliari, Via San Giorgio 12, 09123 Cagliari, Italy

[2] Department of Mechanical, Chemical and Materials Engineering, University of Cagliari, Via Marengo 2, 09123 Cagliari, Italy

[3] Department of Life and Environmental Sciences, University of Cagliari, 09042 Cagliari, Italy

***Correspondence:** giacomo.fais@unica.it; alessandro.concas@unica.it.

Supplementary figures

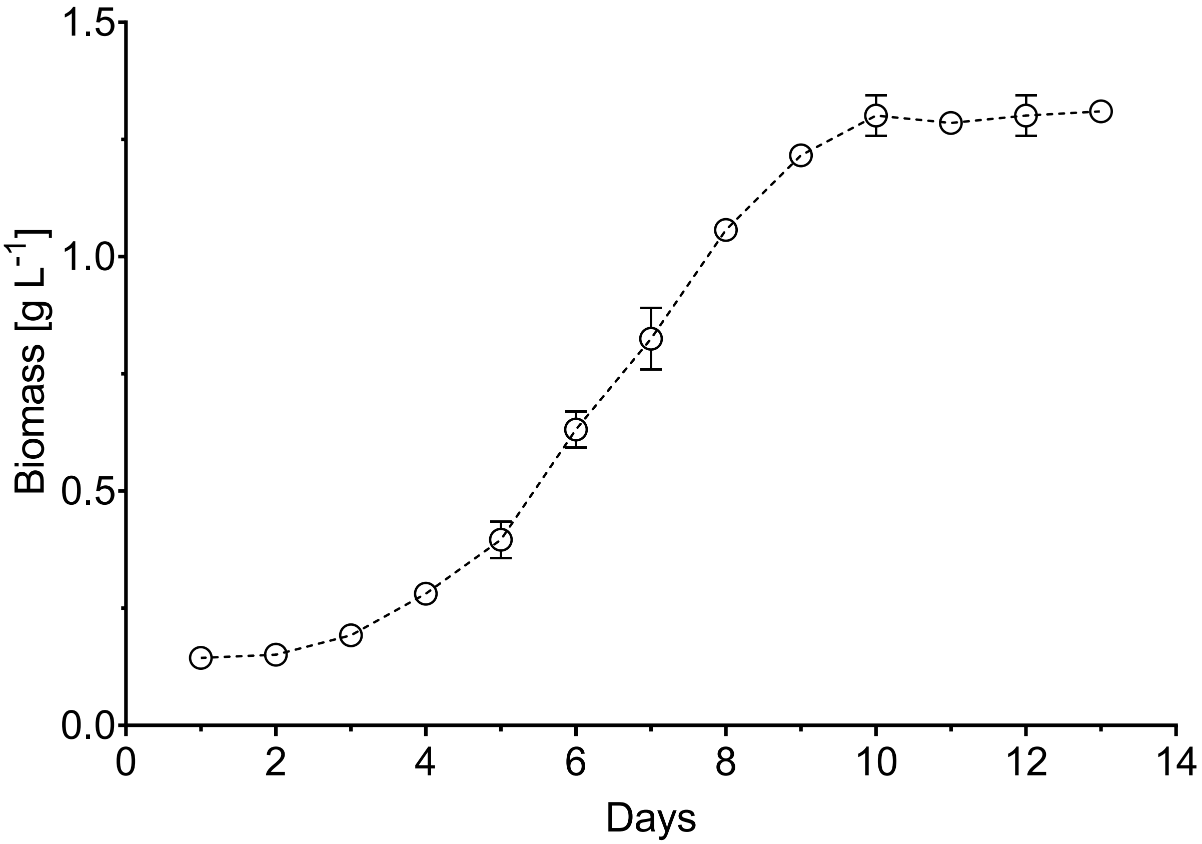

Supplementary Figure 1. Dry weight (DW) biomass profile of *Chlorella vulgaris* over time. Data values are mean ± SD.

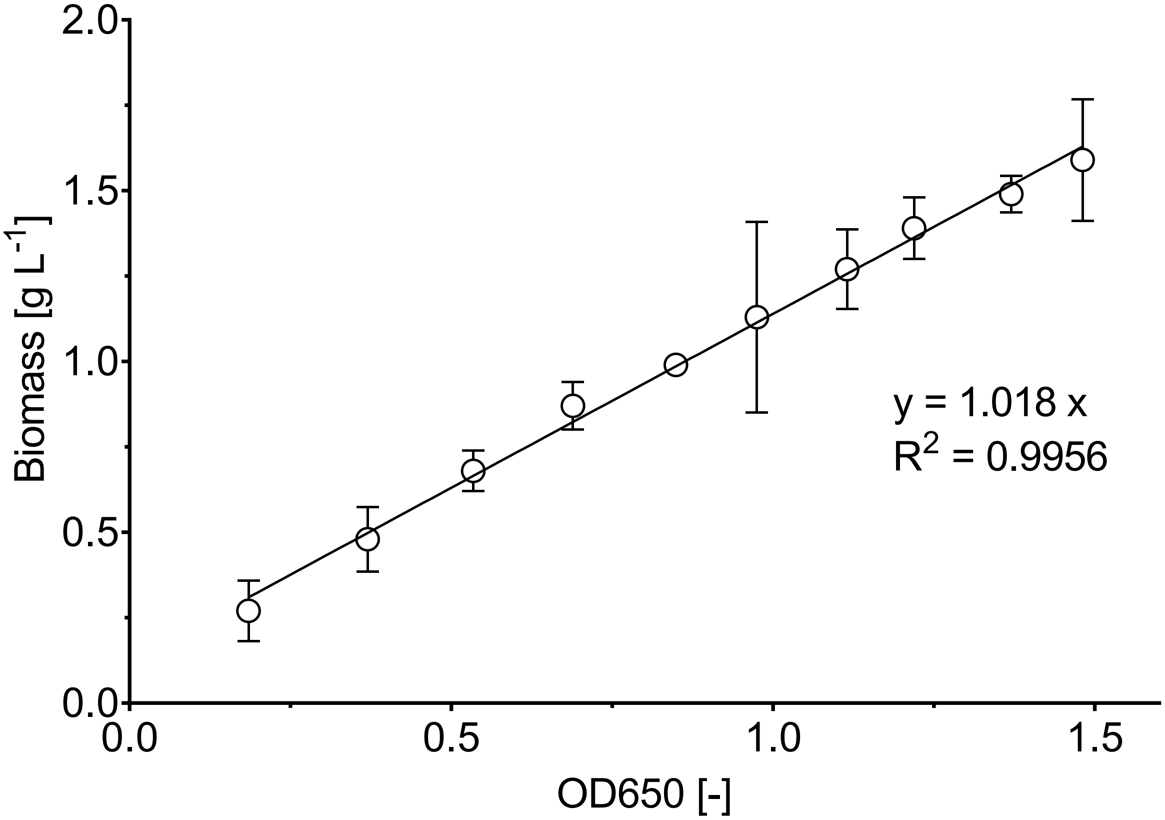

Supplementary Figure 2. Calibration curve of *Chlorella vulgaris* growth: dry weight (DW) biomass (g L^{-1}) vs. absorbance at 650 nm (OD650). Data values are mean ± SD.

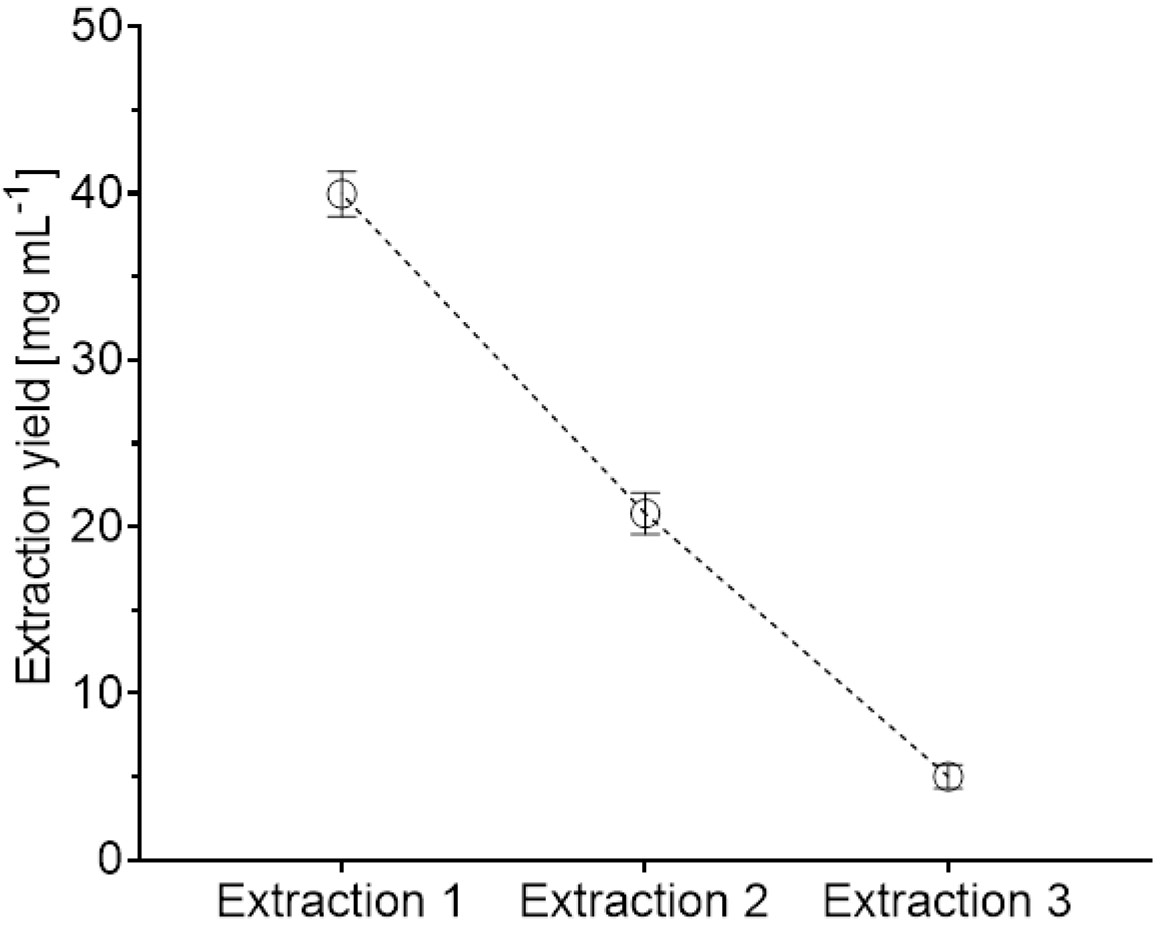

Supplementary Figure 3. Yield of the polar fraction (CEW) from *Chlorella vulgaris* biomass obtained in three sequential Folch extraction cycles. Yield values correspond to the residue concentrations that refer to the dry weight content in 1 mL of polar phase (mg mL^{-1}) and are reported as mean ± SD (n = 4).

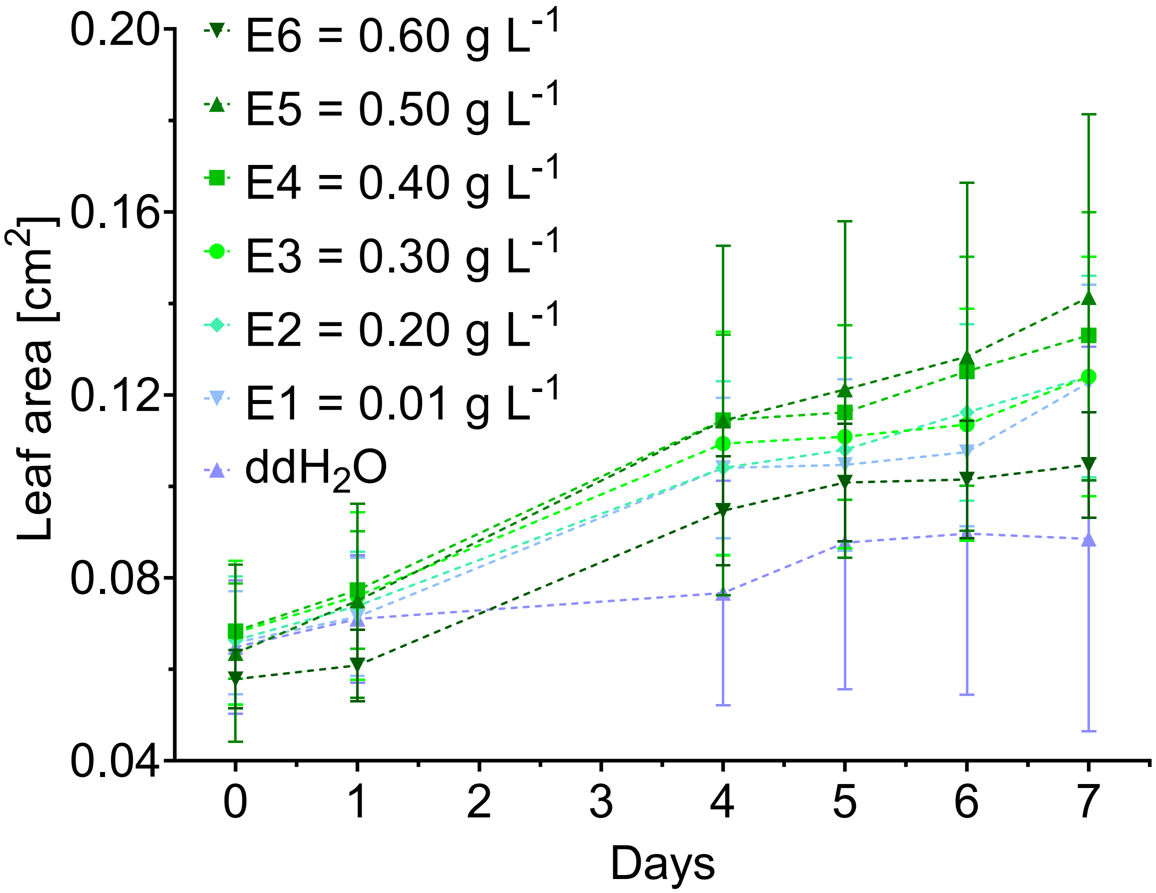

Supplementary Figure 4. Time profile of frond leaf area (cm^2) of *Lemna Minor* under ddH_2O and different concentrations of CEW over 7 days. Data values are mean ± SD (n = 6).

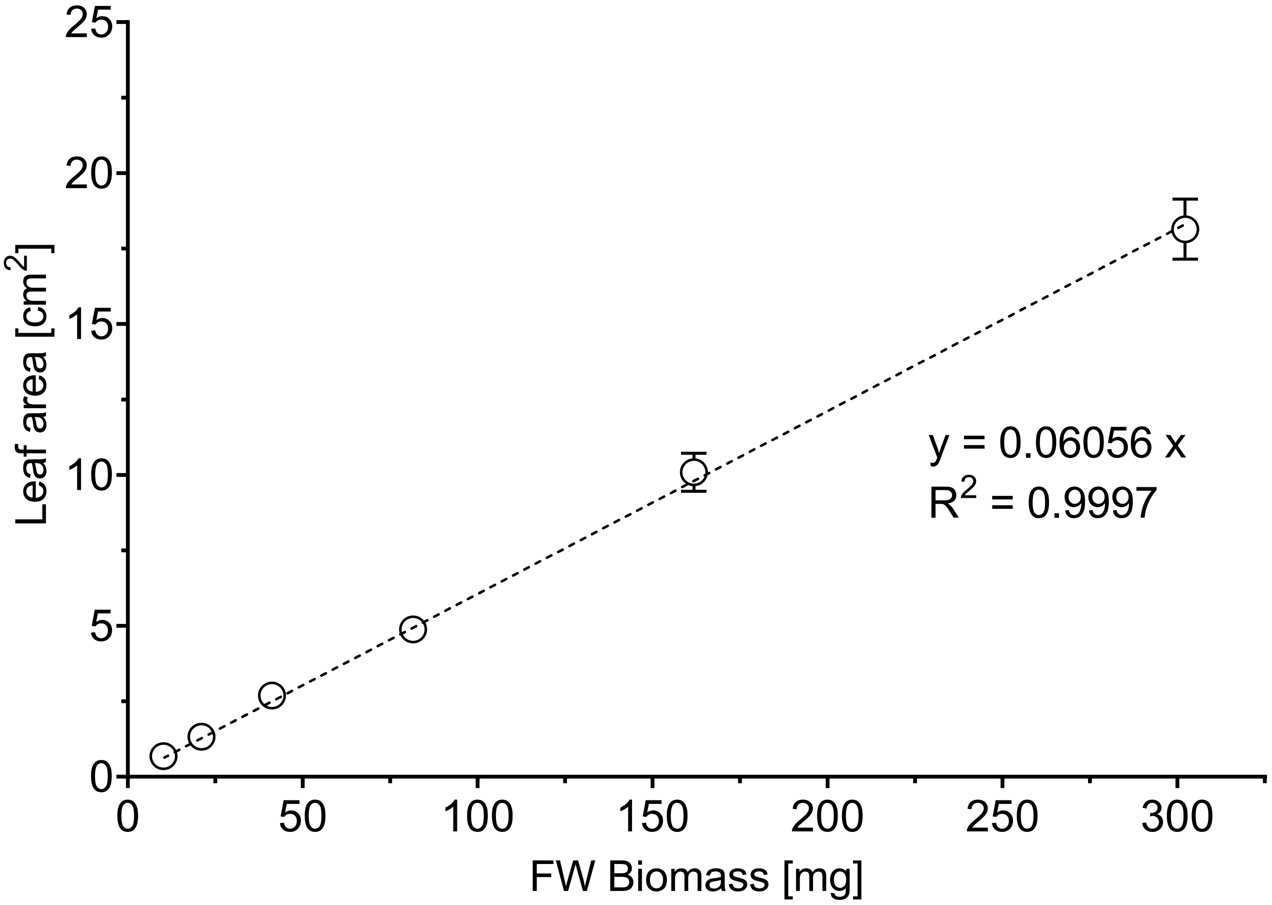

Supplementary Figure 5. Calibration curve of *Lemna minor* growth: leaf area (cm^2) vs. fresh weight (FW) biomass (mg). Data values are mean ± SD.
